# Comprehensive Network Analysis Reveals Alternative Splicing-Related lncRNAs in Hepatocellular Carcinoma

**DOI:** 10.3389/fgene.2020.00659

**Published:** 2020-07-15

**Authors:** Junqing Wang, Xiuquan Wang, Akshay Bhat, Yixin Chen, Keli Xu, Yin-yuan Mo, Song Stephen Yi, Yunyun Zhou

**Affiliations:** ^1^Department of General Surgery, Ruijin Hospital, Shanghai Jiao Tong University School of Medicine, Shanghai, China; ^2^Department of Mathematics and Computer Science, Tougaloo College, Jackson, MS, United States; ^3^Department of Oncology and Department of Biomedical Engineering, University of Texas at Austin, Austin, TX, United States; ^4^Department of Computer and Information Science, University of Mississippi, Oxford, MS, United States; ^5^Department of Neurobiology and Anatomical Sciences, University of Mississippi Medical Center, Jackson, MS, United States; ^6^Department of Pharmacology and Toxicology, University of Mississippi Medical Center, Jackson, MS, United States; ^7^Department of Data Science, University of Mississippi Medical Center, Jackson, MS, United States

**Keywords:** long non-coding RNAs (lncRNA), alternative splicing, multi-graphic random walk, gene-regulatory network analysis, random walk, hepatocellular carcinoma, integrative network analysis

## Abstract

It is increasingly appreciated that long non-coding RNAs (lncRNAs) associated with alternative splicing (AS) could be involved in aggressive hepatocellular carcinoma. Although many recent studies show the alteration of RNA alternative splicing by deregulated lncRNAs in cancer, the extent to which and how lncRNAs impact alternative splicing at the genome scale remains largely elusive. We analyzed RNA-seq data obtained from 369 hepatocellular carcinomas (HCCs) and 160 normal liver tissues, quantified 198,619 isoform transcripts, and identified a total of 1,375 significant AS events in liver cancer. In order to predict novel AS-associated lncRNAs, we performed an integration of co-expression, protein-protein interaction (PPI) and epigenetic interaction networks that links lncRNA modulators (such as splicing factors, transcript factors, and miRNAs) along with their targeted AS genes in HCC. We developed a random walk-based multi-graphic (RWMG) model algorithm that prioritizes functional lncRNAs with their associated AS targets to computationally model the heterogeneous networks in HCC. RWMG shows a good performance evaluated by the ROC curve based on cross-validation and bootstrapping strategies. As a conclusion, our robust network-based framework has derived 31 AS-related lncRNAs that not only validates known cancer-associated cases MALAT1 and HOXA11-AS, but also reveals new players such as DNM1P35 and DLX6-AS1with potential functional implications. Survival analysis further provides insights into the clinical significance of identified lncRNAs.

## Introduction

Alternative splicing (AS) events are frequently observed in tumorigenesis and serve as cancer-driving genes. AS can originate from somatic mutations that disrupt splicing regulatory mechanisms or influence the expression levels of splicing factors or transcription factors (Climente-Gonzalez et al., 2017). Hence, AS-associated genes are recognized as important signatures for tumorigenesis and are of significance in developing therapeutic targets for cancer clinical trial. For example, the SF3B1-targeting compound spliceosome inhibitor E7107 has been implemented in advanced tumor treatment (Eskens et al., 2013).

Studies from Zhang et al. (2016) and Romero-Barrios et al. (2018) showed that long non-coding RNAs [generally more than >200 nucleotides (nt) in length] are associated with a variety of AS mechanisms. lncRNAs may interact with specific alternative splicing factors (ASF) or with other intermediate molecules that affect chromatin remodeling to fine tune the splicing of target genes (Romero-Barrios et al., 2018). For instance, our previous experimental study showed that MALAT1 regulated the ASF, SRSF1 (SF2) in gastric cancer cells (Wang et al., 2014; West et al., 2014). In addition, Ji et al. (2014) reported that MALAT1 promoted tumor growth and metastasis in colorectal cancer through the binding of SFPQ in order to release the oncogene PTBP2. On the other hand, LINC01133 has been reported to interact with splice factor SRSF6 in patients suffering from colorectal cancer (Kong et al., 2016) and non-small cell lung cancer (NSCLC) (Zang et al., 2016).

Proteins that have multiple splicing regulators and that promote the transformation of target genes generally get triggered by transcriptional factors (TFs). For example, the transcription regulator MYC, induces upregulation of hnRNP A1/2, that, in turn, regulates alternative splicing events in expressing the cancer-associated pyruvate kinase M2 (PKM2) isoform (David et al., 2010; Koh et al., 2015). Since lncRNAs occur specifically during pre-transcriptional or post-transcriptional modifications, effectors (such as miRNAs, TFs, or ASFs) that are away from their targets, act as cofactors or guides to alter TF-promoter interactions.

Although studies have identified the correlation of lncRNAs and AS to be important in cancer prognosis, there still remains gaps within current studies as only a few cancer-related AS events are known to be regulated by lncRNAs. In addition, it was not clear how the lncRNAs were linked to specific AS sites, hence, providing no evidence to correlate clinical outcomes. Next-generation sequencing technologies have helped identify ∼40K novel lncRNAs cancer, whose regulatory functions in AS remain unknown in tumorigenesis. Hence, computationally predicting novel lncRNAs and associated alternative splicing events may help in the comprehensive understanding of the HCC disease at a systems level.

In this study, we established an innovative technology for propagating molecular networks called the random walk-based multi-graphic (RWMG) model. The RWMG model simultaneously integrates sophisticated biological connections among lncRNA targets [such as transcription factors (TF), alternative splice factors (ASF), and microRNAs] based on both biophysical interaction networks and their co-expression profiles within a single analytical framework. When comparing conventional random walk algorithms that considers equal proportion of all input genes, our flexible and scalable method can be formulated to rank a subset of lncRNAs based on literature survey. In addition, the method we propose has better accuracy than other previously defined “shortest path” network-based algorithms, with advantages of overcoming “noise” and “incomplete” dimensional heterogenicity from the data.

In addition, previous published reports on comparing tumor and normal tissues are generally limited to normal adjacent tissues (NAT). However, these tissues are not truly “normal” as they are usually surrounded by tumor contaminations. Therefore, many potential cancer biomarkers involved in AS may be missed. Hence, to increase the performance of such analysis, we combined healthy liver tissue samples that were downloaded from GTEx along with expression data from TCGA.

## Materials and Methods

### Data Description and Project Design

The framework of the underlying biological hypothesis and model assumption for this project is described in Supplementary Figures S1A,B. The analysis in this manuscript relied on using multi-omics data. We downloaded gene expression data for 110 normal liver samples from the GTEx and TCGA along with clinical information for 369 liver tumors and 50 normal samples from the UCSC Xena database^1^. The sequencing platform for obtaining gene expression was Illumina HiSeq 2000, and pre-processing of raw data was done following the UCSC’s Xena Toil (Vivian et al., 2017) method in order to quantify gene and transcript isoform expression. Annotation of coding and non-coding genes was obtained using GENCODE v23 (Harrow et al., 2012).

### Identification of HCC Tumor Non-coding Genes (lncRNAs or Pseudogenes) From TCGA and GTEx RNA-seq Data

We performed a method of trimmed mean of *M*-values (TMM) normalization for RNAseq data (Robinson and Oshlack, 2010) so that the expression level for lncRNAs and pseudogenes are comparable. The TMM normalized data was further transformed to log2-counts per million for our linear model. HCC differentially expressed (DE) lncRNAs and pseudogenes between tumor and normal samples (T/N) were analyzed by R package limma (Smyth, 2005) with a statistical cutoff (*p* < 1.0E-04 and fold-change > 2). The identification of DE miRNAs had been reported in our previous work (Wang et al., 2018). The identified HCC-specific expressed features (lncRNAs, pseudogenes, and miRNAs) are expected to represent potential key mechanisms in liver neoplasm.

### Analysis of Alternative Splicing Isoforms and Functional Consequence

In order to analyze alternative splice isoforms, we first discarded those isoforms that contained nil values on abundance levels across all the samples. We used R package “IsoformSwitchAnalyzeR” to analyze individual isoform switches from T/N comparison and their biological processes (Vitting-Seerup and Sandelin, 2017, 2018). Differentially switched isoforms between T/N were determined by the following criteria: difference in isoform fraction (dIF) > 0.1 and FDR-corrected *q*-value < 0.05. The functional consequences of switched isoforms were further analyzed for protein-coding potential (CPAT) (Wang et al., 2013), Nonsense-mediated decay (NMD) status, protein domains (Pfam) (Finn et al., 2015; Potter et al., 2018), and open reading frames (ORF). We used the cutoff 0.364 as suggested to distinguish coding and non-coding isoforms in CPAT analysis. On the other hand, NMD is a process that recognizes mRNAs carrying a premature termination codon (PTC) and that triggers their degradation in order to prevent the synthesis of dysfunctional proteins. AS that controls expression of genes is an important process facilitating mRNA degradation in specific isoforms and could lead to NMD (Cuccurese et al., 2005). Since exon structure of all isoforms in a given gene are with isoform switching capabilities, we obtained their corresponding spliced nucleotide sequence and corresponding coding sequence from ORF positions (Weischenfeldt et al., 2012). The alternative splicing (AS) patterns of switching isoforms were predicted by spliceR (Vitting-Seerup et al., 2014) to include alternative 3′ acceptor sites (A3), alternative 5′ donor sites (A5), exon skipping (ES), mutually exclusive exons (MEE), AS at TF start sites (ATSS), AS at termination site (ATTS), and intron retention (IR). Gene enrichment analysis of features that compared normal vs. tumor samples were performed by following the statistical testing of Fisher’s exact-test. *P*-values were corrected for multiple testing using the Benjamin–Hochberg scheme with an FDR < 0.05.

### Construction of AS-Associated lncRNA Epigenetic Regulatory Interaction Subnetworks in HCC

We collected physical interaction information of lncRNAs and associated targeted genes through database searching and text mining. These interactions were evidenced from experimental validations, neighboring gene pairs, gene fusions, and co-occurrence of lncRNAs that connect with miRNA-, TF-, ASF-, and switched genes. Furthermore, HCC lncRNA-target networks were compiled from the following resources: Chiu et al. (2018), miWalker2.0 (Dweep and Gretz, 2015), STARBASE v2 (Li et al., 2013), and lncRNA-disease (Bao et al., 2018) that were analyzed from several high-throughput assays, including ENCODE enhanced version of the crosslinking and immunoprecipitation assay (eCLIP) and chromatin immunoprecipitation sequencing (ChIP-seq) data (Consortium, 2004). HCC-specific miRNA-target networks have been described in our previous published results (Wang et al., 2018); TF-target predicted interaction networks were manually curated from the following databases and publications: Chiu et al. (2018) (Supplementary Table S5), HTRIdb (Bovolenta et al., 2012), Whitfield (Whitfield et al., 2012), and TRANSFAC (Matys et al., 2006) that were based on combined evidence from ENCODE ChIP-Seq assays and positioned weighted matrix (PWM) for TF motif analysis.

Features that were enriched in AS regulatory pathways were collected from pathCards (Belinky et al., 2015), KEGG spliceosome (Kanehisa and Goto, 2000), NCBI Biosystems mRNA processing (Geer et al., 2009), REATOME mRNA splicing pathway, and processing of capped intron-containing pre-mRNA pathway (Croft et al., 2010). These features were involved in an essential component of splicing factors or non-snRNA spliceosome required for the second catalytic step of pre-mRNA splicing. Among these collected 335 splicing regulator genes, 86 were experimentally validated as alternative splicing factors (ASF). ASF and target gene interactions were manually confirmed from SpliceAid 2 (Giulietti et al., 2012), ASF motif analysis from SFmap (Paz et al., 2010), a subset of RNA-binding protein network by Chiu et al. (2018) (Supplementary Table S6), and STRING database (Franceschini et al., 2012).

Finally, identified HCC-DE lncRNAs, pseudogenes, and miRNAs were mapped to the global regulatory networks to construct HCC-specific sub-networks that contain switched genes as the targets or TF/ASF as the co-effectors of non-coding RNA regulators.

### Construction of HCC lncRNA-AS Regulatory Networks at Isoform Level

Pearson correlation was used to estimate the lncRNA co-expression relationships at isoform level. We only included connections for the pairs of lncRNA and protein-coding genes, with absolute correlation coefficient greater than 0.75 and FDR *p* < 0.05. The types of protein were either TFs, ASFs, or genes with isoform switches. lncRNAs that were negatively correlated with their targeted protein-coding genes were predicted to be inhibitors, while positive correlation indicated activators.

### Random Walk Multi-Graphic Model for the Integration of Heterogeneous Interaction Networks

Random walk multi-graphic (RWMG) model is an integrative application of random walk with restart (RWR) algorithm on multiple layers of heterogeneous network. Our framework is encoded with data sets for the same cohort of patients including:

(1) Co-expression network, which is a bipartite graph containing the association between *n* lncRNA and *l* AS genes.
(2) Epigenetic regulatory network, which is also a bipartite graph containing the association between *n* lncRNA and *k* AS genes (*p*≠ l). Note: the Epigenetic regulatory network and Co-expression network share the same set of *n* lncRNA nodes, but the AS genes of the two networks are partially distinct.
(3) Splicing pathway PPI networks, which is an *m × m* AS gene–AS gene interaction network with *m* nodes. The node set is the union of distinct AS genes from the Co-expression and Epigenetic regulatory networks with size *m*. There is no information about interaction between lncRNA.

We first create an extended graph *G*(*V, E_*k*_*) with *N nodes* for each given network, where *V* is the union of *n* lncRNA and *m* AS gene nodes and *N = n + m, k = 1,2,3*, which represent co-expression, epigenetic, and splicing pathway PPI networks, respectively. In addition, these were merged into one undirected association network *MG*(*V*,*E*),*E* = U*E*_*k*_. Multiple edges are allowed to connect between any two nodes based on the relationship defined from networks. Merged network with the overlapped node features and the union of edges will augment each individual network with missing connections. We let *A* denote the adjacency matrix of a (weighted) molecular interaction multi-graph network *MG*(*V, E*). Edge (*i*, *j*) ∈ *E*,1≤*i*,*j*≤*N* is weighted by the connectivity score between these vertices. The connectivity score

$$E_{i,j}=\frac{\sum_{k=1}^{3}{\left[E_{k}\right]}_{i\,j}}{3}$$

is the average of all included edge scores connecting nodes *i,j*. It is the edge weight to shape the adjacent matrix *A*.

Each entry *B*_*ij*_ in the transition probability matrix *B*, which stores the probability of a transition from node *j* to node *i*, is computed as

$$B_{i\,j}=\frac{A_{i\,j}}{\sum_{k=1}^{N}A_{k\,j}}$$

Therefore, we can write the RWMG model on a multi- and heterogeneous- graph *MG*(*V, E*) as:

$$p^{t+1}=\left(1-\alpha \right)\,B\,p^{t}+\alpha \,p_{s}$$

where vectors *p*^*t* + 1^*and p^t^* are *N*-dimensional column vectors where *p*^*t*^[*i*] denotes the probability of being at node *i* and *t* iteration, and α is the probability of restart (we set α = 0.5 in this paper). *p*_*s*_ is an *N*-dimensional column vector with *n* lncRNA and *m* AS gene with *p*_*s*_ (seed) = 1 and others are 0. After a restart step, the particle can go back either to a seed lncRNA feature or to a seed AS gene. We implemented the RWR algorithm on the final multi-graphic network by R package dnet and igraph (Csardi and Nepusz, 2006; Fang and Gough, 2014). Network visualization was performed by R package visNetwork (Guerrieri, 2015). Those genes with known roles in regulating AS network will be set as the “seed” nodes in advance to predict the “new” lncRNAs, based on move probabilities from the current node to any of their randomly selected neighbors.

To evaluate our approach’s sensitivity, we simulated different random walk strategies for optimization. We created a list of experimentally validated AS-associated genes as “gold-standard” true positive genes (TPG) curated from the careful literature review and randomly selected genes as the “gold-standard” true negative (TNG). We chose the “best” model that has the most candidates significantly enriched in the “gold-standard” gene list. In reality, the number of TPG is much smaller compared to TNG. To avoid bias from highly imbalanced data between these two sets, we performed a bootstrap resampling technique by selecting an equal number of data as TNG. This process was repeated 10 times, and the overall performances were calculated by the mean value of these performances.

### Survival Analysis for Prognostic Confirmation of Identified Pathogenic lncRNAs and Pseudogenes

To confirm the pathogenic characteristics of identified lncRNAs and pseudogenes, univariate Cox proportional model was used to evaluate the association of selected genes with overall survival outcomes. Kaplan–Meier plots and log-rank test statistics were used to visualize the high- and low-risk groups. The cutoff of the high- and low-risk group was determined by the median value of the normalized count of selected genes.

## Results

### Differentially Expressed lncRNAs and Pseudogenes in HCC

**FIGURE 1:**
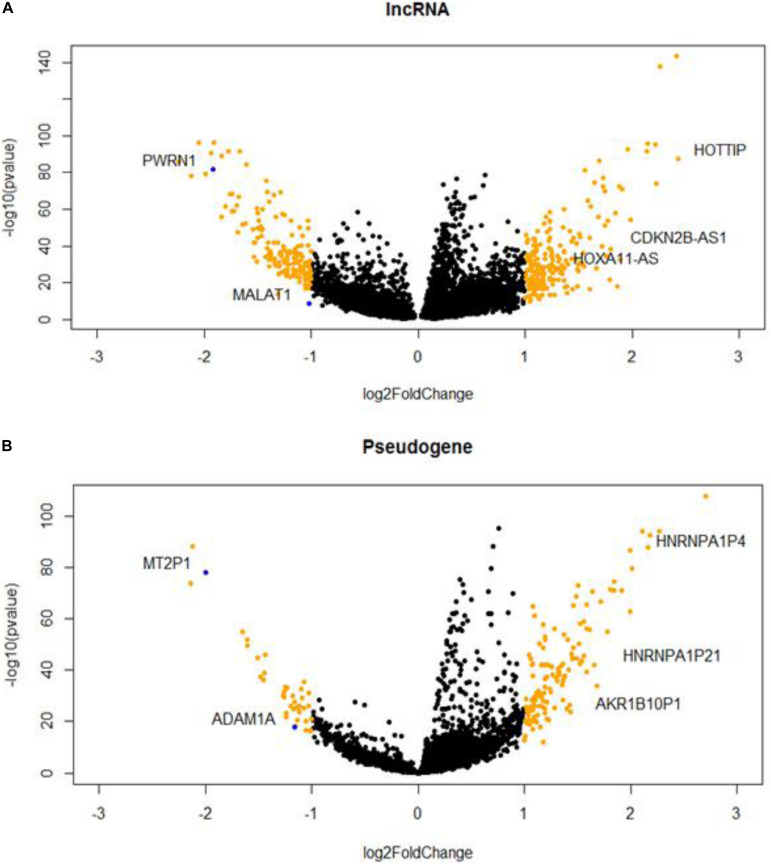
Hepatocellular carcinoma (HCC)-specific long non-coding RNAs (lncRNAs) **(A)** and pseudogenes **(B)** that are differentially expressed in tumor and normal samples.

We identified 369 DE lncRNA genes and 171 DE pseudogenes from T/N comparison (Supplementary Table S1). The visualizations of DE lncRNAs and pseudogenes were shown in volcano plots (Figure 1). According to literature survey, many DE lncRNAs, such as MALAT1, CDKN2B-AS1, and HOTTIP, have been reported to be associated with liver cancers (Kunej et al., 2014; Quagliata et al., 2014; Guerrieri, 2015). In addition, we highlighted several important pseudogenes, such as HNRNPA1P4 and HNRNPA1P21, which are heterogeneous nuclear ribonucleoproteins A1 (hnRNPs) that play key roles in the regulation of alternative splicing. Furthermore, we performed DE analysis as an initial screen step to narrow the focus of the HCC-specific non-coding genes associated with AS for the downstream network analysis.

### Identification of Significant Switched Isoforms and Prediction of Alternative Splicing Patterns

**FIGURE 2:**
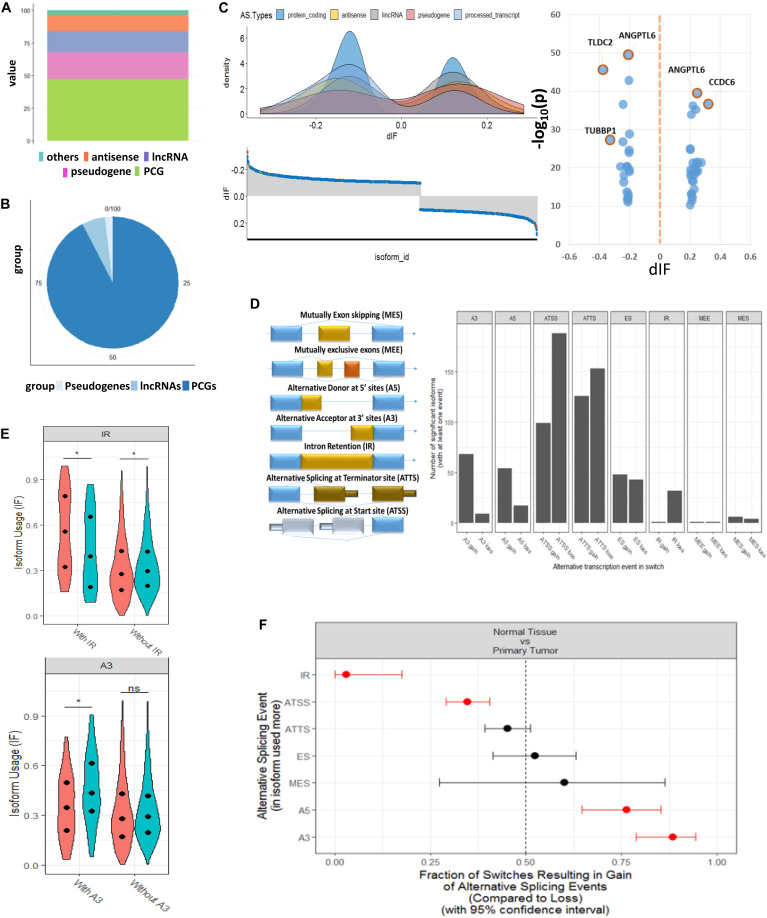
Genome-wide transcript analysis for switched isoforms between tumor vs. normal comparison HCC. **(A)** Global distribution of whole genome transcriptions based on GENCODE annotation. The percentage of coding and non-coding genes is about half and half. **(B)** Distribution of the HCC-switched isoforms in coding and non-coding region. About 95% of switched isoforms are from protein-coding genes. **(C)** Distribution of differential isoform fraction (dIF) stratified by coding or non-coding isoform types. The most significantly switched isoforms (dIF > 0.2) are highlighted. **(D)** Illustration of alternative splicing event types for the switched isoforms and distribution of isoform gain (increased dIF) or loss (decreased dIF) in each types. **(E)** Enrichment analysis for alternative splicing types in isoform fraction gain or loss. Intron retention (IR) and alternative splicing at termination site (ATSS) categories are enriched in loss switches, while A5 and A3 are significantly enriched in gain. **(F)** Distribution of dIF changes with or without IR and A3 events. Isoforms showed less usages in IR type and more usage in A3 type.

From the expression levels of isoform when comparing tumor and normal samples, we identified 1,375 isoforms that had switching properties and that mapped to 1,078 unique genes. Among these switched isoforms, 1,251 were protein-coding isoforms, and 124 were non-coding isoforms that included antisense, lncRNA and pseudogenes (Supplementary Table S2). We found that the proportion of switching rate for coding genes was much higher than that for non-coding genes (Fisher’s exact test, *p* = 8.4e-08) (Figures 2A,B). In order to visualize the splicing composition of these switched isoforms, we broke down the dIF distribution according to isoform types such as lncRNA, antisense, and pseudogenes with the most significant switched isoforms (dIF > 0.2 or dIF < -0.2) highlighted in Figure 2C.

Figure 2D shows the eight splicing patterns for switched isoforms stratified by isoform usage gain or loss in the tumor. Some of the switched isoforms are predicted to have multiple AS events in HCC (Supplementary Table S3). Interestingly, we observed a global phenomenon that the AS events are not equally used—most prominently illustrated by the use of ATSS in HCC, where there was more losses than the gain of amino acid coding exons. It should be taken into consideration that IR and ATSS were enriched in significant low isoform usage in tumor, but A5 and A3 were significantly enriched in the gain isoform (Figure 2E). Here, IR events were of particular functional interest since they represented the largest changes in isoforms. As we show in the violin plots, the enriched IR and A3 splicing groups reported significant opposite directions of isoform usages between T/N samples (Figure 2F).

### Analysis of Functional Consequences for Switched Isoforms

**FIGURE 3:**
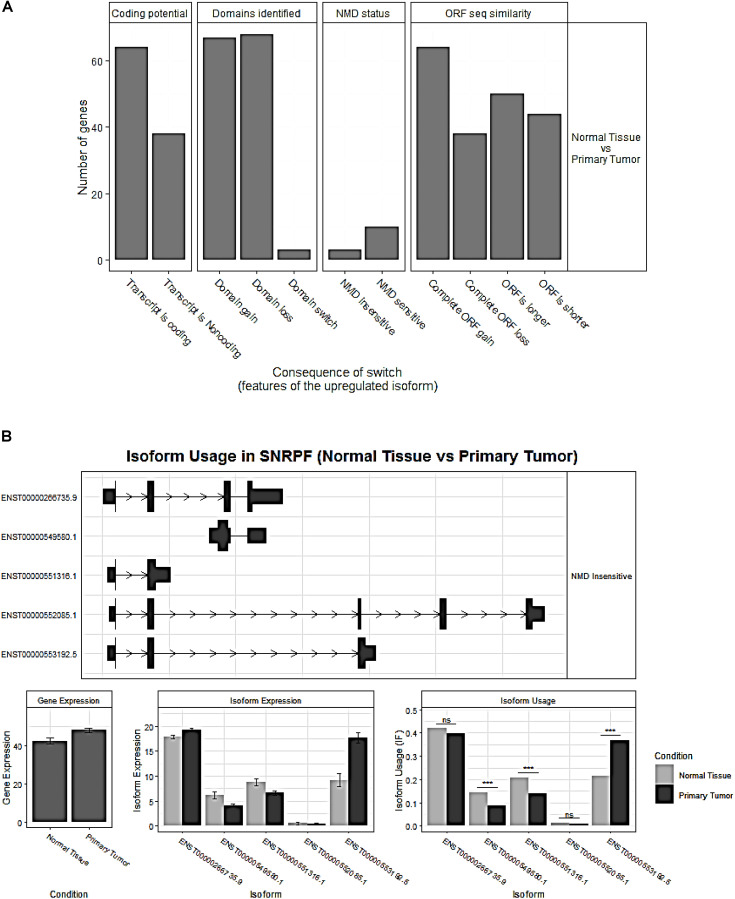
**(A)** Overview of the number of switched isoforms predicted to have functional consequences. **(B)** Visualization of switched isoform structure. Taking a splicing factor gene, SNRPF, for example, its isoform ENST00000553192.5.1 showed opposite switching pattern compared to others. In addition, three out of five isoforms showed differential isoform expressions, although no difference for the overall gene expression.

The overview of switched isoforms impacting the biological function alterations in HCC is shown in Figure 3A. The number of protein domain gains was comparable to domain loss, but is significantly more than domain “switch.” Here, the “switch” term indicates both a gain and a loss occurrence. Also, switching resulting in ORF gain was significantly more than ORF loss. For the Gene Ontology analysis, both gain and loss switched isoforms were associated with different types of metabolic processes. KEGG analysis showed that the isoform loss in tumor tissue was associated with virus infection, hepatitis C, etc, while isoform gain in the tumor is associated with base excision repair, apoptosis, etc. (Supplementary Table S2).

Importantly, we confirmed 20 genes with switched isoforms that were involved in AS regulatory functions (Table 1). Figure 3B shows one example of AS factor, SNRPF and its isoform structures, gene expression, and isoform usage when comparing tumor vs normal. SNRPF is a core component of U small nuclear ribonucleoproteins that are key components of the pre-mRNA processing spliceosome. We found no significant difference for SNRPF gene expression; on the contrary, it had opposite directions in expression pattern for transcript ENST00000553192. The above evidences showed that genes with switched isoforms were often functionally important in tumorigenesis and had been ignored from previous reports.

**TABLE 1:** Statistic summary of splicing factor genes with alternative switched isoforms.

**Isoform_id**	**Gene_id**	**Gene_name**	**dIF**	**q_value**
ENST00000555295.1	ENSG00000100836.10	PABPN1	0.182	1.10E–32
ENST00000459687.5	ENSG00000100410.7	PHF5A	0.172	6.07E–18
ENST00000411938.1	ENSG00000128534.7	LSM8	0.169	2.49E–19
ENST00000553192.5	ENSG00000139343.10	SNRPF	0.152	6.22E–21
ENST00000297157.7	ENSG00000164610.8	RP9	0.145	2.68E–19
ENST00000491106.1	ENSG00000060688.12	SNRNP40	0.128	4.28E–19
ENST00000560313.2	ENSG00000090470.14	PDCD7	0.124	2.17E–06
ENST00000301785.5	ENSG00000214753.2	HNRNPUL2	0.116	1.19E–28
ENST00000402849.5	ENSG00000100028.11	SNRPD3	0.113	1.67E–11
ENST00000535326.1	ENSG00000110107.8	PRPF19	0.103	1.79E–07
ENST00000597776.1	ENSG00000130520.10	LSM4	0.102	2.31E–34
ENST00000472237.5	ENSG00000132792.18	CTNNBL1	0.102	5.80E–13
ENST00000548994.1	ENSG00000075188.8	NUP37	0.101	1.68E–11
ENST00000564651.5	ENSG00000102978.12	POLR2C	0.1	3.01E–14
ENST00000505885.1	ENSG00000096063.14	SRPK1	–0.108	1.94E–11
ENST00000404603.5	ENSG00000100028.11	SNRPD3	–0.109	2.30E–15
ENST00000540127.1	ENSG00000214753.2	HNRNPUL2	–0.116	2.99E–48
ENST00000367208.1	ENSG00000182004.12	SNRPE	–0.13	1.79E–31
ENST00000527554.2	ENSG00000100697.14	DICER1	–0.139	2.98E–21
ENST00000595761.1	ENSG00000213024.10	NUP62	–0.157	3.62E–31
ENST00000488937.1	ENSG00000136875.12	PRPF4	–0.159	6.94E–12
ENST00000559051.1	ENSG00000090470.14	PDCD7	–0.163	9.60E–15
ENST00000216252.3	ENSG00000100410.7	PHF5A	–0.216	4.30E–21

### Prediction of AS-Correlated Non-coding RNAs at Both Transcript and Gene Level

**FIGURE 4:**
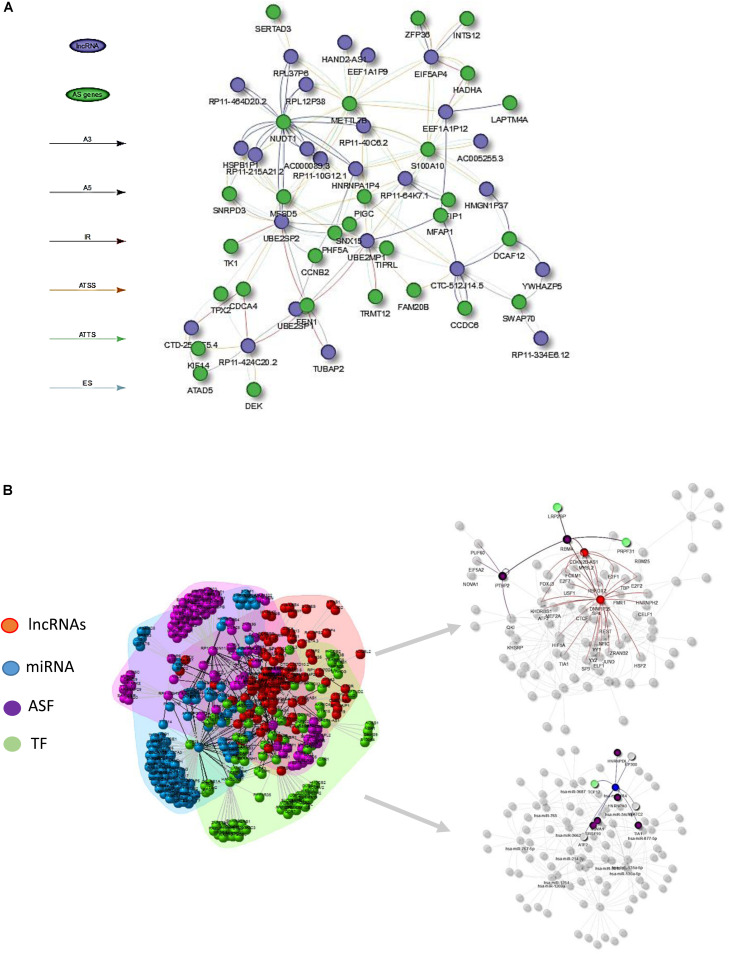
**(A)** Visualization of lncRNA–AS co-expression network integrated by AS event types (i.e., A3, IR, ES) at the isoform expression level. **(B)** Illustration of lncRNA–AS comprehensive network derived from gene level co-expression network and regulatory network involved with co-effectors miRNA, TF, and alternative splicing factors (ASF) interactions.

In order to identify which lncRNAs were associated to switched isoforms at the transcript level, we constructed a co-expression network that comprised lncRNA and genes with switched isoforms. Different from traditional gene level co-expression network, the connections between lncRNA and genes with multiple splicing isoforms could be singular or multiple when interacting between molecules. The lncRNAs-switched isoform connections are summarized in Supplementary Table S3. The relationships between lncRNAs and genes with enriched AS patterns is illustrated in Figure 4A.

However, since the lncRNA regulation mechanism involved in AS events was comprehensive, AS regulation may not directly be reflected from expression abundance, but through physical interaction or DNA/RNA binding sites. LncRNAs could influence gene-splicing patterns by inhibiting and activating the expression of ASFs, or through transcription factors that indirectly interact with splicing factors and ultimately cause changes in AS factor-targeted gene expression. Hence, constructing a comprehensive gene regulatory network that includes TF, AS regulators, and lncRNAs could allow better understanding of the mechanism of AS in cancers.

Figure 4B illustrates the HCC lncRNAs–AS network with interactors such as TFs, ASFs, and miRNAs based on evidence from publicly available resource and gene-level co-expression analysis. Only lncRNAs that directly altered AS gene expression or indirectly altered AS genes through TF, ASF, or miRNAs were included for downstream RWMG analysis. Supplementary Table S4 provides the prediction of all AS-related genes ranked by RWMG-predicted score. Supplementary Table S6 provides the total number of nodes and edges for the three types of networks.

### Computational and Clinical Validation for Predicted Pathogenic lncRNAs Involved in AS Regulation

**FIGURE 5:**
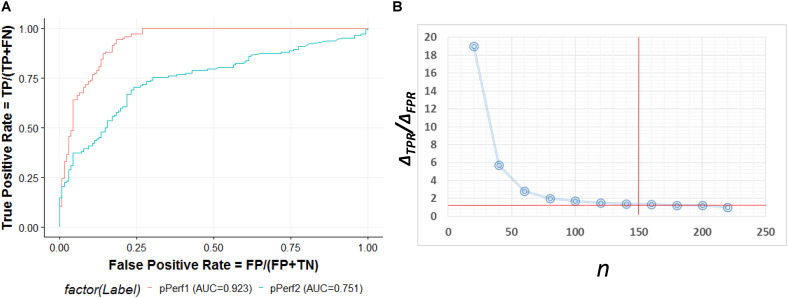
**(A)** ROC curve for the predictive model evaluation. pPerf1 [area under curve (AUC) = 0.923] with the “seed” genes showed a better performance than pPerf2 (AUC = 0.751) without the “seed” genes. **(B)** Trade-off between the sensitivity and specificity with the number of top *n* genes. We can see that the best cutoff is *n* = 150, as the ΔTPR/ΔFPR value decreases very fast in the beginning and approaches smaller changes for n around 150.

The ROC curve shown in Figure 5A contains an optimized averaged area under curve (AUC) value from 0.751 to 0.923 based on bootstrapping algorithm. In order to select the best number of top *n* ranked genes that corresponded to a fair tradeoff between sensitivity and specificity, we selected a cutoff based on the trend of the changes at Δ*TPR*/Δ*FPR* that exhibited a sudden drop (Figure 5B). We also see from the figure that *n* = 150 is the best number for selecting genes. The top ranked lncRNAs associated with AS functions are described in Table 2.

**TABLE 2:** Statistic summary of predicted top-ranked non-coding RNAs associated with alternative splicing (AS) ranking by random walk-based multi-graphic (RWMG) score.

**Gene. symbol**	**Ranking**	**Score**	**Types**
LINC00675	1	0.00368174	LincRNA
CTD-2171N6.1	2	0.002824633	LincRNA
HOTTIP	3	0.002677841	Antisense
DNM1P35	4	0.002483954	Antisense
LEF1-AS1	5	0.002397948	Antisense
AP006285.7	6	0.002123275	LincRNA
WARS2-IT1	7	0.002081091	Antisense
LINC00355	8	0.002063983	LincRNA
RP11-81H3.2	9	0.002032482	LincRNA
HOXA11-AS	10	0.002028198	Antisense
RP11-261N11.8	11	0.002005615	Antisense
RP3-355L5.4	12	0.001938399	Antisense
RP11-138J23.1	13	0.001929433	LincRNA
RP11-525K10.3	14	0.001923733	Antisense
RP11-495P10.7	15	0.001917542	LincRNA
DLX6-AS1	16	0.00188837	Antisense
RP11-356C4.5	17	0.001861006	LincRNA
CDKN2B-AS1	18	0.001856714	Antisense
RP11-495P10.5	19	0.001834267	LincRNA
SFTA1P	20	0.001751498	LincRNA
PRSS51	21	0.001750058	Antisense
MALAT1	22	0.001672339	LincRNA
FEZF1-AS1	23	0.001669135	Antisense
RP4-530I15.9	24	0.001619806	Antisense
RP11-158M2.5	25	0.001618054	Antisense
CTD-2374C24.1	26	0.001617345	LincRNA
PWRN1	27	0.001605646	LincRNA
CTC-573N18.1	28	0.001534221	LincRNA
RP11-284F21.9	29	0.001527715	LincRNA
RP11-3J1.1	30	0.001523171	LincRNA
FENDRR	31	0.001509286	LincRNA

**FIGURE 6:**
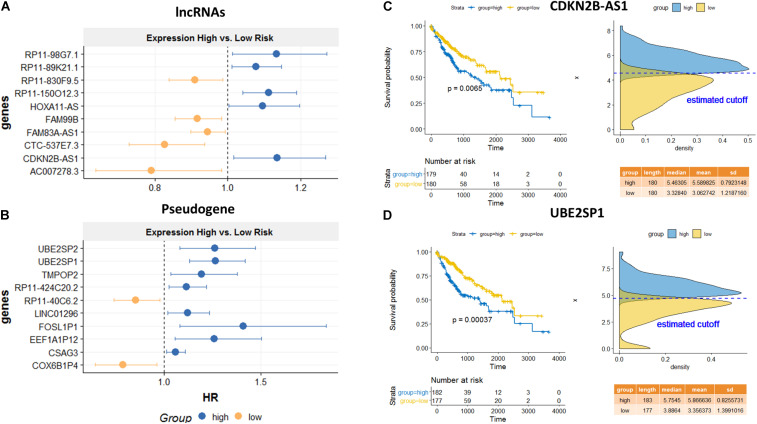
Survival analysis for the identified lncRNA and pseudogenes involved in AS mechanisms. Hazard ratio plots from Cox regression analysis for top 10 lncRNAs **(A)** and top 10 pseudogenes **(B)** associated with overall survival. K–M curves for a lncRNA, CDKN2B-AS1 **(C)**, and a pseudogene, UBE2SP1 **(D)** using the median value as the cutoff. High expressions of both genes are significantly associated with poor prognosis.

Among the top predicted lncRNAs that were involved in AS, we further confirmed their clinical significance. As a result of univariate survival analysis screening, a total of 51 lncRNAs and 24 pseudogenes were found to be associated with HCC overall 5-year survival, respectively (Supplementary Table S5). Figures 6A,B show the top 10 significant genes based on the Cox proportional regression model. Figures 6C,D show the survival curve and distribution of CDKN2B-AS1 and UBE2SP1.

## Discussion

In the last decade, studies have investigated the association of splicing isoforms and lncRNA profiles from deep sequencing technologies. For instance, it has been known that some small nuclear uridine (U)-rich RNAs (snRNPs) are core components of the pre-mRNA processing spliceosome and can collaborate with some splicing factors that are encoded by heterogeneous nuclear ribonucleoprotein complex subunits (hnRNPs) in order to fine tune complex splicing regulations (Romero-Barrios et al., 2018). Impressively, we found a number of core snRNP isoforms including SNRPE, SNRPD3, SNRPD3, SNRPF, and SNRNP40 that were switched even though their expression was not necessarily DE when comparing tumor vs. normal specific to HCC progression. SNRNP40 catalyzes the removal of introns from pre-messenger RNAs. Similarly, an hnRNP U like protein HNRNPUL2 that also has a scaffold attachment factor, plays an important role in the formation of a “transcriptional” complex binding through the scaffold attachment region and causes chromatin remodeling.

The primary mechanisms involving lncRNAs in AS modulation can be classified into three ways that include: (i) lncRNAs that directly influence isoform expression through activation or inhibition mechanism; (ii) lncRNAs that form RNA–RNA duplexes with pre-mRNA molecules, and (iii) lncRNAs that affect target AS genes through indirectly inhibiting or promoting the expression of splicing factors or through transcript factors. However, most previous studies only focus on individual genes and/or isoform switches regulated by lncRNAs. More comprehensive interactions can be detected at the isoform level besides the gene level. Our predictions identified several candidates that were either oncogenes or tumor suppressors and lncRNAs whose somatic alterations were associated with AS at both isoform and gene level in addition to showing clinical significance in HCC patients.

At the transcriptional level correlation network, we found that majority of lncRNA isoforms were correlated with more than one AS event, among which some were showing opposite roles in the AS regulations. In addition, we can see that many lncRNAs may partially compete with the same AS event. For example, the pseudogenes of UBE2S, which are UBE2SP1, UBE2SP2, and UBE2MP1, are significantly correlated with FEN1’s intron retention and Alternative 5′ donor site mechanisms (Figure 4A). The FEN1 gene plays an important role in removing 5’ overhanging flaps and the 5–3 exonuclease activities involved in DNA replication and repair (Wang et al., 2017), while the UBE2S is involved in ubiquitination and subsequent degradation of VHL, which results in an accumulation of HIF1A (Jung et al., 2006). However, the reason for pseudogenes being associated with FEN1 is not yet clear. Further research in regard to perform experimental validation for predicted mechanisms from our analysis is necessary. Taken together, these results confirmed that the identified lncRNAs need to be better investigated in experimental settings. Our results provided a better resolution of AS-correlated lncRNAs at the isoform level.

AS events are mainly regulated by splicing factors, which bind to pre-mRNAs and influence exon selection and splicing site choice. Moreover, TFs activate or suppress the expression of ASF. Importantly, we found ASF that may have switched isoforms. A switched ASF RP9, which can be bound by the proto-oncogene PIM1 product, a serine/threonine protein kinase, also can cause its target PIM1 to get switched. Although TFs were usually thought for a long time to encode a single protein that changes the expression of their target genes, more and more TFs are now found to be alternatively spliced (Marcel and Hainaut, 2009). Here, we also found a group of TFs in the ETS family (E26 transformation-specific), which are ETS1, ETS2, ETV3, ELF4, which were switched simultaneously. These ETS genes have been confirmed to be associated with cancer through gene fusion (Tomlins et al., 2005) and are involved in a wide variety of regulatory functions such as cell migration, proliferation, and cancer progression (Sharrocks, 2001; Lee et al., 2005). Interestingly, the ETS1 targets splicing factor QKI, and ETV3 targets splicing factor CELF1. Furthermore, lncRNA FAM99B is predicted to be associated within the ETS family genes, and their low expression is associated with HCC patients that had poor prognosis.

The association of CDKN2B-AS1, also known as ANRIL, with HCC has been reported in several studies (Hua et al., 2015; Chiu et al., 2018; Ma et al., 2018). CDKN2B-AS1 has both linear and circular isoforms, and their functions are different. For example, its linear isoform can regulate the c-myc-enhancer-binding factor RBMS1 (Hubberten et al., 2018), while its circular isoform is confirmed to be an important AS regulator that causes skipping of exons (Holdt et al., 2016) and are mainly found in cardiovascular disease (Burd et al., 2010; Sarkar et al., 2017). However, this is the first time we found that ANRIL can activate alternative splicing genes in liver cancer. A potential explanation could be because of being functionally related to lipid metabolism and a majority of liver cancer subtypes. In addition, the prognostic value of CDKN2B-AS1 was revealed in our project. However, how exactly CDKN2B-AS1 controls this gene splicing is not yet clear. Further experimental validation is warranted. We identified HAND2-AS1 gene to show consistent alternative splicing pattern at the start sites and termination site for METTL7B especially at the isoform level. METTL7B is a membrane-associated protein that resides in hepatic lipid droplets. An explanation for this is that HAND2-AS1 activates the METTL7B spliced isoform lipid disordered and is associated with HCC, which was not reported before. Gene-level RWMG network analysis further revealed that both CDKN2B-AS1 and HAND2-AS1 can influence AS either through TFs and ASFs some of which include HAND2-AS1 TFs (i.e., ETS1, SP1, E2F7) or ASFs (i.e., SRSF7, SFRP1, HNRNPK); and CDKN2B-AS1-associated TFs (SP4, E2F7) and ASF (SRSF1, SRSF2).

In this project, we extended a previous existing algorithm into multiplex and heterogeneous networks. The research community can explore different layers of the epigenetic regulatory network, expression correlation network, and protein interaction network. A recent Nature Review paper by Cowen et al. (2017) also suggested that the “network-propagation” method was a “powerful” and “accurate” refined approach in the network biology, since it is capable of dealing with “noise” and “incomplete” observations by simultaneously considering all possible paths among vertices. Analyzing these heterogeneous data together will significantly improve the prediction accuracy of our method. By using this gene-ranking strategy, potentially spurious predictions (false positives) that are supported by a single (shortest) path are down-weighted, and true high-ranked genes that are potentially missed, even though they are well connected to the prior list (false negatives), are promoted.

To our best knowledge, this is the first attempt to predict lncRNA regulations on AS using a rigorous, multi-graphic approach by the integration of large-scale and complex networks. Of interest for potentially limiting the accuracy of random walk and network propagation methods are an incomplete collection of known lncRNAs, especially pseudogenes, used to supervise prediction of new candidates. As such, we addressed several unique challenges associated with these dataset complexities in each step. For example, in the data preprocessing steps, we carefully address the challenges by collecting as many as experimentally verified and predicted lncRNAs that were taking account of AS. In our statistical modeling steps, we specifically addressed the robustness of complex data integration, especially for non-informative or noisy datasets. Also, we investigated several random walk strategies by trying different groups of vertices such as lncRNAs, ASFs, and TFs as a starting point to optimize our models.

However, the lncRNA regulatory mechanism is complicated, as its mechanism differs with different stages, such as the pre-mRNA or post-mRNA stage. Therefore, the major limitation of this article is we were not able to consider other comprehensive mechanisms at different stages, such as recognition of the splicing site can be modulated by *cis*-regulatory sequences, known as splicing enhancers or silencers, which contribute to the generation of two or more alternatively spliced mRNAs from the same pre-mRNA. Also, lncRNA determines AS patterns through chromatin remodeling mechanism and shapes the three-dimensional genome organization. We will focus on interpreting these molecular mechanisms of lncRNA and associated AS at different stages of HCC in the near future.

## Data Availability Statement

Publicly available datasets were analyzed in this study. TCGA and GTEx data can be found in data hubs in https://xenabrowser.net/.

## Author Contributions

JW contributed to the analysis and interpretation of data. XW contributed to mathematics model interpretation. AB reviewed and edited the manuscript. AB and SY contributed to data analysis, provided feedback, and edited the manuscript. YC and KX contributed to the machine learning predictive model design. YM contributed to the project design and interpretation of biological meanings. YZ led the project, provided the guidance, and prepared the manuscript. All the authors read and approved the manuscript.

## Conflict of Interest

The authors declare that the research was conducted in the absence of any commercial or financial relationships that could be construed as a potential conflict of interest.

## Footnotes

1 http://xena.ucsc.edu/

## Supplementary Material

FIGURE S1 **(A)** Illustrations of overall project design, and **(B)** explanation of biological mechanisms.

TABLE S1 Statistic summaries for significantly differential expression genes between tumor and normal comparison for lncRNAs and pseudogenes.

TABLE S2 Statistic summaries and functional analysis for significantly switched isoforms between tumor and normal comparison for lncRNA and protein-coding genes, as well as prediction of splicing event patterns for switched genes. Gene ontology and KEGG pathway enrichment analysis are performed for upregulated isoforms (gain) and downregulated isoforms (loss), respectively.

TABLE S3 Predicted lncRNA interaction pairs at the transcriptional co-expression level.

TABLE S4 Statistic summary of AS-associated lncRNAs and PCGs ranking by RWMG predictive score.

TABLE S5 Statistic summary of significant lncRNAs and pseudogenes associated with overall survival.

TABLE S6 Total number of node and edge distribution for the three networks.
