# Supplementary figures and images for: Comprehensive Network Analysis Reveals Alternative Splicing-Related lncRNAs in Hepatocellular Carcinoma
Source: Front Genet. 2020 Jul 15;11:659. doi: 10.3389/fgene.2020.00659 (PMC7373802; doi:10.3389/fgene.2020.00659)

Figure S1 (A)

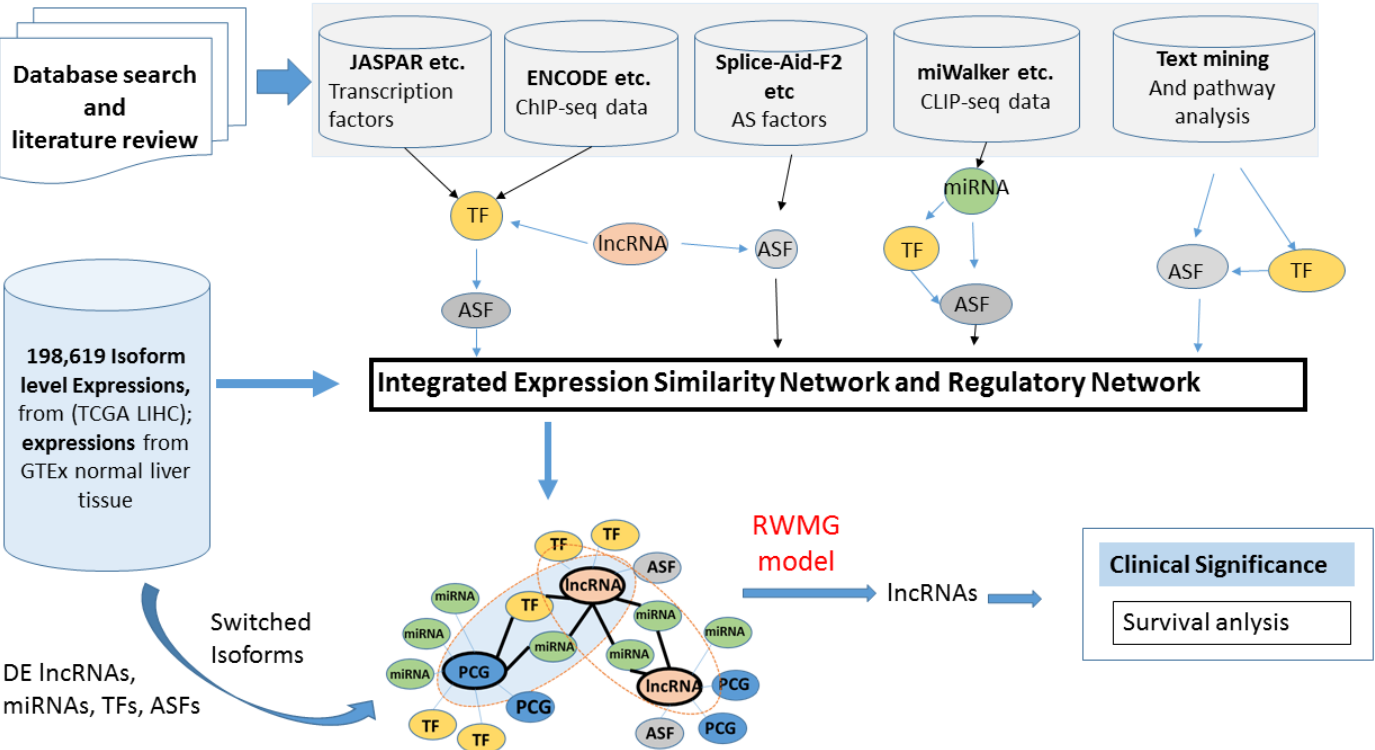

(B)

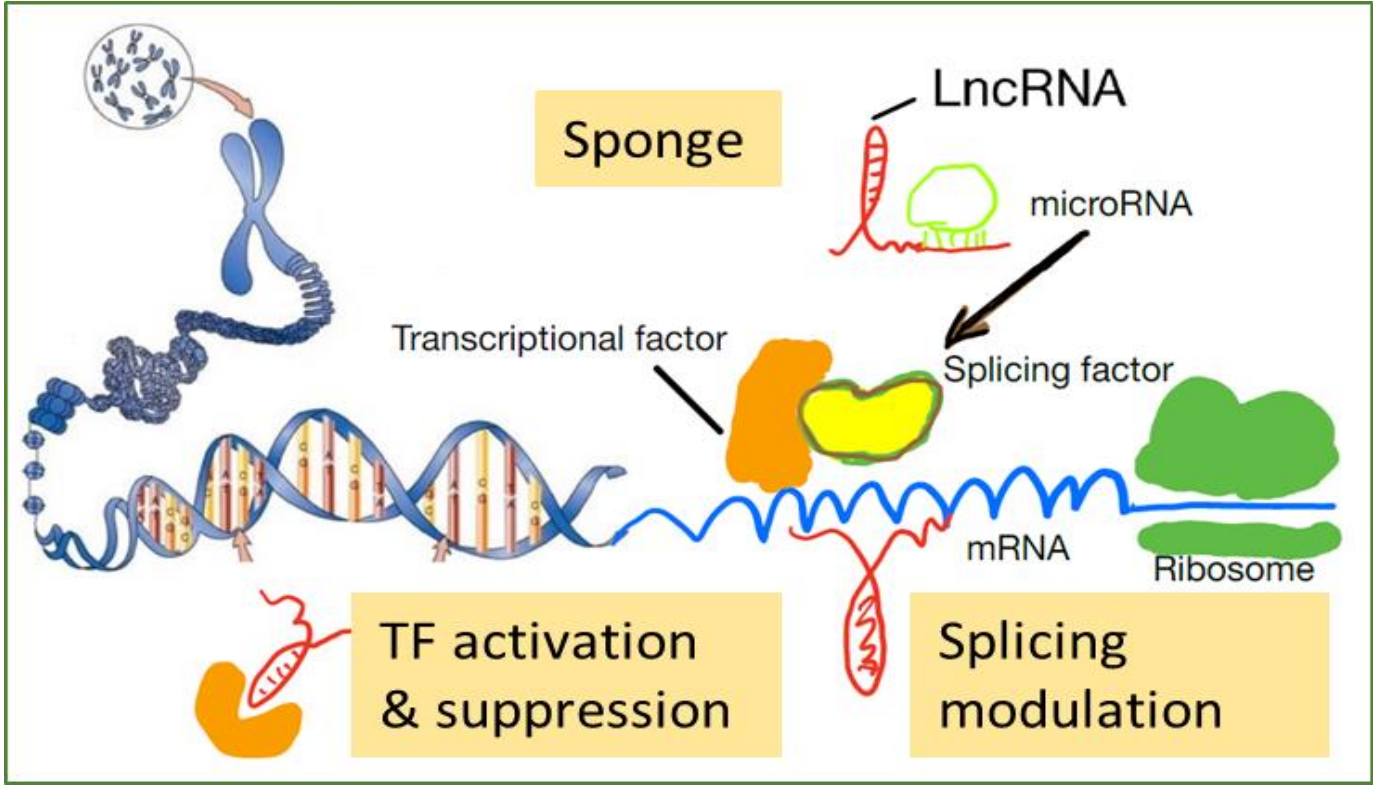

Supplement: FIGURE S1 — (A) Illustrations of overall project design, and (B) explanation of biological mechanisms. [file Image_1.pdf]
